# [Retracted] HCV‑E2 inhibits hepatocellular carcinoma metastasis by stimulating mast cells to secrete exosomal shuttle microRNAs

**DOI:** 10.3892/ol.2024.14434

**Published:** 2024-05-08

**Authors:** Li Xiong, Shuqing Zhen, Qionghua Yu, Zuojiong Gong

Oncol Lett 14: 2141–2146, 2017; DOI: 10.3892/ol.2017.6433

Following the publication of this paper, it was drawn to the Editor's attention by a concerned reader that certain of the Transwell migration and invasion assay data shown in Fig. 4D on p. 2145 were strikingly similar to data that had already been published in different form in another article written by different authors at a different research institute [Feng L, Ma J, Li H, Liu Y and Hu W: MiR-184 retarded the proliferation, invasiveness and migration of glioblastoma cells by repressing stanniocalcin-2. Pathol Oncol Res 24: 853–860, 2018].

Owing to the fact that the contentious data in the above article had already been published prior to its submission to *Oncology Letters*, the Editor has decided that this paper should be retracted from the Journal. The authors were asked for an explanation to account for these concerns, but the Editorial Office did not receive a reply. The Editor apologizes to the readership for any inconvenience caused.

